# Full‐scale slurry tank sampling: Effects of sampling strategy and sample storage on measured physical and chemical properties

**DOI:** 10.1002/jeq2.70128

**Published:** 2025-12-22

**Authors:** Jesper Nørlem Kamp, Morten Kjærulff Sørensen, Johanna Pedersen

**Affiliations:** ^1^ Department of Biological and Chemical Engineering Aarhus University Aarhus Denmark; ^2^ NanoNord A/S Aalborg Denmark

## Abstract

Accurate analyses of liquid animal manure (slurry) from full‐scale slurry storage tanks are essential for effective nutrient management in agriculture. Inadequate or inconsistent sampling methods and improper storage conditions can lead to significant inaccuracies in nutrient analysis of slurry. This study evaluates the impact of different slurry sampling methods, storage types, and durations on the physical and chemical properties of slurry. Slurry was sampled using three different sampling methods in the same full‐scale tank both before and after mixing. Samples were stored at 5°C and −18°C for different durations (1–24 weeks) to assess changes in physical and chemical properties of the slurry. The analyzed parameters were ammonium, total nitrogen, total phosphorus, sodium, dry matter, volatile solids, and pH. The results show that the measured parameters varied considerably with sampling methods, in some cases by up to 80%–100%. The largest differences were observed for dry matter and total phosphorus between top and bottom samples in the unmixed tank, reflecting sedimentation effects. Profile sampling gave similar results as mixing prior to sampling. Storage at 5°C maintained sample integrity over 4 weeks, while storage at −18°C led to substantial changes in pH. Our findings indicate that proper sampling is crucial for representative analysis and that storage conditions can influence pH over extended periods.

## Core Ideas

- Liquid animal manure (slurry) composition varies significantly with sampling depth, highlighting the need for proper sampling procedures.
- Profile sampling in an unmixed tank can provide representative slurry samples.
- The change of slurry pH during storage emphasizes the importance of measuring pH directly at the tank.
- Standardized sampling and storage are essential for accurate slurry analysis.

## Plain Language Summary

Farmers use slurry (liquid animal manure) to fertilize crops, but testing slurry for nutrients can be tricky. The results depend on how the slurry is sampled and stored before it is tested in a lab. Slurry often separates over time, with heavier solids sinking to the bottom. This study looked at how different ways of collecting slurry samples—and storing them before testing—affect the accuracy of the results. The study showed that mixing the tank before sampling makes the nutrients more evenly spread. But even without mixing, a good sample can be taken by collecting from top to bottom and mixing the sample afterward. It was also found that freezing slurry samples can change the results, especially the pH (a measure of acidity). Accurate slurry testing helps farmers apply the right amount of nutrients to their fields. This supports better crop growth and reduces pollution. The results can help improve how slurry is sampled and stored, leading to better nutrient management on farms.

**Abbreviations**
CV: coefficient of variation
DM: dry matter
NMR: nuclear magnetic resonance
slurry: liquid animal manure
TN: total nitrogen
TP: total phosphorus
VS: volatile solids

## 1 INTRODUCTION

Liquid animal manure (slurry) is utilized as a crop nutrient in agriculture. The composition of slurry depends on factors such as animal diet, bedding material, and storage type and conditions. Slurry is a heterogenous liquid with suspended particles varying in size and numbers, resulting in large differences in slurry viscosity (Thygesen et al., 2012).

Information about chemical and physical properties of the slurry can be used by the farmer for management decisions for field application of the slurry, with the aim of increasing crop yield while minimizing nutrient losses and environmental impact (Fangueiro et al., 2015; Hafner et al., 2018; Smith et al., 2008). For optimal utilization of slurry nutrients for crop production, accurate knowledge of the nutrient concentrations (Pandey et al., 2018; Suarez‐Tapia et al., 2018) and other chemical properties and physical characterization (Fangueiro et al., 2017; Pedersen et al., 2022) is necessary. Additionally, some countries have regulations determining an upper limit for certain nutrients (e.g., nitrogen and phosphorus) that can be applied per area unit (BEK No 1102, 2022; Schröder & Neeteson, 2008), making it necessary to know the concentration of these in the slurry prior to application. The accuracy of the slurry analysis is limited by how representative the slurry sample is; thus, the sampling strategy is highly important because inaccuracies caused by sampling or sample storage can result in over‐ or under‐application of nutrients (O'Dell et al., 1995).

Likewise, accurate concentration measurements are crucial for research and inventory purposes. As an example for research purposes, the measured total ammoniacal nitrogen (NHx‐N, also known as TAN) can be used directly in combination with emission measurements after field application of slurry to determine ammonia emission factors (Hafner et al., 2021; Kamp et al., 2024). Thus, potential inaccuracies in NHx‐N concentrations caused by biased sampling directly cause errors in the emission factors. For international inventory reporting, the Intergovernmental Panel on Climate Change encourages the use of a “Tier 3” inventory calculation (EEA, 2023; IPCC, 2019), which requires country‐specific input data to give accurate emission estimates. As an example, the ALFAM2 model (Hafner et al., 2021, 2025) is used for inventory calculations in Denmark, where slurry parameters are crucial input parameters and have a high effect on the model output (emission factors). Therefore, inaccuracies in determining average slurry values for Denmark will result in biased emission factors for the inventory and hence less accurate emission reporting.

Slurry is usually stored in tanks or lagoons before field application (Kupper et al., 2020). Previous studies have highlighted the significant impact of slurry sampling and storage conditions on the accuracy of obtained slurry nutrient contents. Dou et al. (2001) demonstrated the need for multiple samples from storage, and Dou et al. (2001) and Aguirre‐Villegas et al. (2018) showed that thorough agitation creates a more uniform nutrient distribution in the slurry, resulting in more representative samples and less variability between samples from the same storage. Johannesson et al. (2018) highlighted how slurry stratification in storage, especially without agitation, affects nutrient variability and emphasized that sampling at different depths is necessary for representative results if storage is unmixed. Collectively, these studies stress the importance of proper sampling techniques to obtain reliable slurry nutrient data, particularly since slurry is not always mixed before loading into the slurry trailer. In such cases, profile‐based sampling may provide a more accurate estimation of nutrient contents. Some slurry spreaders are equipped with sensors that continuously measure nitrogen concentration to adjust slurry distribution; however, information about other important variables such as pH and phosphorus is still lacking.

Previous studies have focused on specific aspects of slurry sampling, such as agitation (Aguirre‐Villegas et al., 2018) and sampling location (Johannesson et al., 2018); however, there is a need for a complete slurry sampling and storage strategy in terms of mixing, sampling, and storage. Existing research has examined the benefits of full‐depth profile sampling, which could capture nutrients throughout the entire storage depth to ensure representative samples, but it has not been coupled with storage condition and length. Prior studies have generally only used a single storage type (either refrigeration or freezing), and potential effects of these and the storage durations have not been assessed. This study addresses these gaps by investigating the impact of a complete depth profile compared to grab samples, with and without prior mixing, two storage conditions, and extended storage times, providing insights into slurry sampling and storage strategies.

Obviously, accuracy of the analytical methods applied to characterize the slurry samples is important as well. In this work, we applied the Tveskaeg nuclear magnetic resonance (NMR) sensor (NanoNord A/S), which is a low‐field NMR instrument for quantification of nutrients in collected slurry samples. This allows easy and accurate quantification of NHx‐N, total nitrogen (TN), and total phosphorus (TP), with as high a precision as wet‐chemistry laboratory methods (Jensen et al., 2021). The NMR instrument has been compared to standard laboratory measurements for animal slurry with very good agreement and even better precision for some analytes (Jensen et al., 2021; Sørensen et al., 2015). Our present study includes sodium (Na^+^) analyses as well, as these are rapidly obtained by the Tveskaeg NMR sensor from the same subsample as analyzed for the other nutrients. Quantification of Na^+^ in slurry by Tveskaeg NMR sensor technology has previously been utilized for slurry analysis, for example, by Sørensen et al. (2023) to enable estimation of nitrogen emissions from slurry in naturally ventilated barns.

In the present work the following sampling strategies were investigated: (a) collecting slurry grab samples from multiple locations within the tank and mixing the subsamples into one homogenous sample, (b) taking a profile sample from the full height of the tank, and (c) thoroughly mixing the entire tank before sample collection.

Each method has its challenges: (a) obtaining true grab samples for each position, (b) collecting a full profile sample including the floating layer and sediment, and (c) the impracticality of time requirement and energy consumption for frequent, thorough mixing. Proper mixing is expected to achieve a homogenous mixing of nutrients in the slurry; however, ammonia emissions are increased during mixing, and there is a risk that the sediment is not properly suspended into the liquid if the mixing is not sufficient. Addressing these challenges ensures that samples accurately reflect the overall slurry composition, thereby minimizing sampling biases and improving nutrient management practices. To our knowledge, no previous study has provided a systematic, full‐scale investigation of slurry sampling strategies combined with different storage conditions and durations. This work is therefore important for addressing slurry sampling and storage before chemical analysis.

The aim of this work was to assess how different slurry sampling methods from a full‐scale slurry tank and storage type and duration affect obtained physical and chemical properties of cattle slurry. Our hypotheses were (i) sampling a profile of an unmixed tank provides equivalent results as a mixed tank, (ii) slurry sampling method affects the slurry sample dry matter (DM), NHx‐N, TN, and TP, but does not affect pH, and (iii) storage type and length do not affect the slurry properties.

**Core Ideas**

- Liquid animal manure (slurry) composition varies significantly with sampling depth, highlighting the need for proper sampling procedures.
- Profile sampling in an unmixed tank can provide representative slurry samples.
- The change of slurry pH during storage emphasizes the importance of measuring pH directly at the tank.
- Standardized sampling and storage are essential for accurate slurry analysis.

## 2 MATERIALS AND METHODS

### 2.1 Slurry tank

The slurry tank was 11 m in diameter and 3 m high, whereof 1 m was below ground. It had a concrete roof with two openings and a capacity of approximately 285 m^3^ (Figure S1). The slurry tank was filled over a few days around September 1, 2022, with slurry from dairy barns at the Danish Cattle Research Centre, Aarhus University with a maximum age of 1 week at the time of filling. After filling, slurry was not added and the tank was not mixed, hence the tank was undisturbed from the beginning of September until the samples were taken in the beginning of March. During this period, a thin surface crust formed, which was removed prior to top sampling. This approach reflects common farm practice for farmers with several tanks that often fill one tank after the other or fill satellite tanks rather than adding slurry continuously.

### 2.2 Slurry sampling

The sampling device used for bottom and profile sampling consisted of a PVC tube approximately 3.5 m long with a diameter of 50 mm. At the bottom of tube, a ball valve allowed the operator to open and close the tube to control sample intake (Figure S2). This design enabled collection of either bottom or complete vertical profile samples from the slurry tank. A similar device has been described elsewhere (e.g., Myrbeck et al., 2019).

Slurry was collected from the slurry tank by three different approaches:

1. Top sampling: Slurry was collected from the top using a jug after removing the thin crust.
2. Bottom sampling: Slurry was collected from approximately 0.05 meters above the bottom using the sampling device.
3. Profile sampling: The sampling device was open during submersion and closed upon reaching the bottom to collect a full vertical profile (besides the bottom 0.05 m).

**FIGURE 1:**
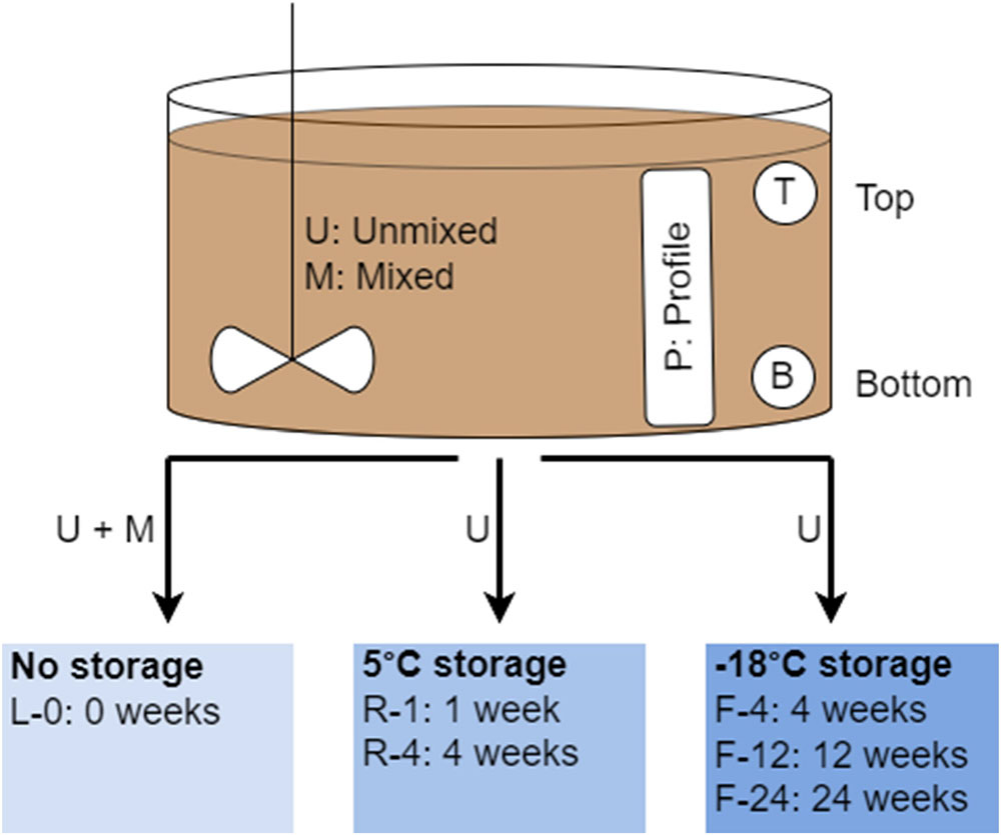
Overview of sampling and storage.

Slurry samples were collected over 3 days (February 27 to March 1, 2023) so that it was possible to analyze some of the samples directly after collection. An overview of the sampling points, storage conditions, and abbreviations used can be found in Figure 1. On the first day, samples of the unmixed tank at unmixed top and unmixed bottom positions were collected, each from three different positions. On the second day, unmixed profile samples were collected from the unmixed tank at three different positions. On the third day, the slurry was mixed with a propeller mixer on a tractor for 4 h, and samples were collected directly after mixing was stopped. Mixed top, bottom, and profile samples were collected for the fully mixed tank from three different positions each. The procedure followed the procedure described by Myrbeck et al. (2019) with sampling for several hours, combining subsamples into a composite sample before homogenizing the sample by mixing while taking the final samples. For all slurry samples, 8–10 L of slurry from the same location was collected in a large bucket. The slurry in the bucket corresponded to one specific name, for example, unmixed top sample 1 (U‐T‐1), which was mixed thoroughly before transferring subsamples to 0.5‐L plastic containers for later laboratory analysis after storage. For example, there were six samples for U‐T‐1 (ID 1–6 in Table S1). A total of 63 samples were collected and analyzed; see Supporting Information for an overview of all slurry samples and positions (Table S1).

### 2.3 Sample storage

Samples were analyzed in the laboratory directly after sampling and after storage in a refrigerator (5°C) for 1 or 4 weeks or storage in a freezer (−18°C) for 4, 12, or 24 weeks. Furthermore, pH was measured immediately after sampling at the tank.

### 2.4 Slurry analysis

An overview of all the analysis and number of repetitions for each analysis can be found in Table S1. The samples from the mixed slurry tank were only measured as fresh samples and not after storage because it was not expected that the samples collected after mixing would differ in composition. The pH was measured in all samples immediately after collection at the tank; thus, there were only 9 pH measurements at the tank for the mixed samples, while there were 54 for the unmixed samples.

Before analysis, slurry samples were blended with a homogenizer (IKA, Ultra Turrax T25 digital homogenizer) with knife assembly (IKA, S25N‐25G, Ø25 mm, 50–2000 mL). The frozen samples were thawed in the refrigerator. After thawing, samples were left at room temperature until they reached a temperature of 20°C–22°C. All samples were prepared and analyzed at 20°C–22°C.

The slurry was analyzed for contents of NHx‐N, TN, TP, and Na^+^ using a Tveskaeg Benchtop NMR instrument (NanoNord A/S) with a 24‐slot automatic sample changer (NanoNord A/S). For each homogenized slurry sample, 2–4 subsamples were aspirated into NMR tubes with 9 mm outer diameter (using NMR tube as a syringe), and each filled tube was closed by pressing the tube onto a plug in a dedicated holder while holding the tube in vertical position. The filled and closed NMR tubes were inserted in the sample changer and measured serially. To quantify concentrations from each subsample, isotopes were measured individually, and the following measuring times were used: 25 min for ^14^N NMR (to quantify NHx‐N and TN), 1 min for ^1^H NMR (for TN), 25 min for ^31^P NMR (to quantify TP), and 2 min for ^23^Na NMR (to quantify Na^+^). Multiple subsamples were taken for NMR to ensure the best practical accuracy of the results, and all results shown are given as the averages of measurements of these subsamples. The number of subsamples for each sample is given in Table S1, and it varies between 2, 3, or 4, since the sample changer was loaded with 24 samples every time measurements were performed.

DM and ash content as a measure of volatile solids (VS) were measured gravimetrically by heating the slurry mixtures to 105°C for at least 24 h in a B180 Oven (Nabertherm) and subsequently burning them at 550°C for 6 h in a Muffle Furnace (Nabertherm) (ASTM E1756‐08 and ASTM E1755‐01). The VS content was reported as a fraction of DM. The pH was measured with a pH electrode (VWR, model DJ 114) after three‐point calibration. The pH was measured directly at the top of the tank before sampling on day 1 and after mixing on day 3. Furthermore, pH was measured in each of the 0.5‐L plastic bottles directly after being transferred and in the laboratory after storage.

When comparing two individual numbers, the relative differences between two numbers were used, and it was calculated as the difference between the numbers divided by the average value of the numbers. The standard deviation and the coefficient of variation (CV) for concentration measurements of the same sample were used to assess the variability.

## 3 RESULTS AND DISCUSSION

**FIGURE 2:**
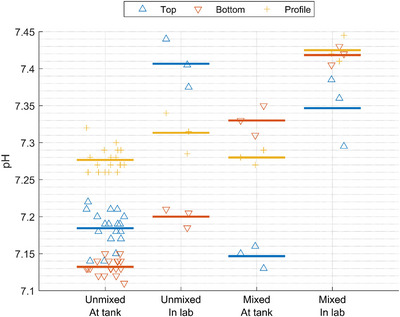
pH measured for mixed and unmixed liquid animal manure (slurry) tank either at the tank or in the laboratory (lab) a few hours after sampling. pH was measured in all samples at the tank after sampling and in the lab only for the fresh samples not undergoing storage. The symbols show individual measurements, and the horizontal line shows the average values.

The pH in the top of the tank (approximately 5‐cm depth) was 7.19 ± 0.03 (standard deviation) on day 1 (before mixing) and 7.17 ± 0.001 on day 3 (after mixing). The average pH measured at the tank from all subsamples was 7.20 ± 0.07 and 7.25 ± 0.09 for the unmixed and mixed samples, respectively. However, variation between the different positions was evident, but the patterns were not similar for the mixed and unmixed tank (Figure 2). The average pH measured in the laboratory a few hours after sampling was 7.40 ± 0.04 for the mixed tank, while the unmixed tank had an average pH of 7.31 ± 0.10. Thus, pH measurements conducted a few hours later in the laboratory of the same subsamples showed an increase in pH of 0.11–0.15 pH units (Figure 2). In both the unmixed and the mixed tank, pH in the top samples measured at the tank and pH measured directly in the top of the tank only differed by 0.01 and 0.02 pH units for the unmixed and mixed situation, respectively. This shows that the mixing itself is not causing any difference for top sampling, but the sampling might cause the increase observed for the profile samples that increased approximately 0.1 pH unit when measured directly at the tank. This is likely due to carbon dioxide emissions causing a pH increase (Hafner et al., 2013) when the profile sample is added to a bucket for subsampling and pH measurements. This would be interesting in future studies measuring carbon dioxide directly. Nonetheless, the pH is very similar between profile samples from the mixed and unmixed tank.

**FIGURE 3:**
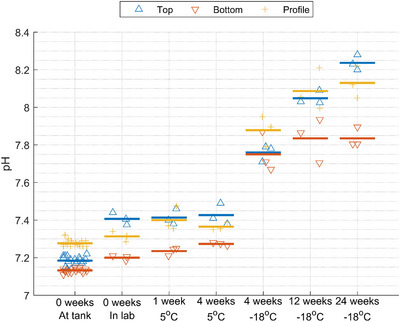
pH measurements in samples from an unmixed liquid animal manure (slurry) tank either at the tank or in the laboratory on the day of sampling. Laboratory measurements of samples stored in the refrigerator (5°C) for 1 or 4 weeks and stored in a freezer (−18°C) for 4, 12, or 24 weeks. The symbols show individual measurements, and the horizontal line shows the average values.

Samples stored at 5°C showed minimal pH changes over 1–4 weeks (<0.1 pH units) compared with pH measured in the laboratory before storage (Figure 3). In contrast, pH increased 0.3–0.5 pH units during storage at −18°C for 4 weeks compared to samples stored at 5°C for 4 weeks, indicating that storage type and not length is causing the difference. The pH increased further over time, with the highest pH observed after 24 weeks at −18°C (Figure 3), where pH increased by >1.0 pH unit during storage. pH should ideally be measured directly at the experimental site during measurements or in the field before/during slurry application, as it can change rapidly due to changes in surface conditions and volatilization of carbon dioxide, ammonia, and volatile fatty acids (Hafner et al., 2013). If it is necessary to store slurry samples, pH should ideally be measured prior to the storage period, or the potential pH changes during storage should be considered. The change in pH during freezing is likely due to mineral precipitation and dissolution during the freeze‐thaw cycle, affecting the chemical equilibriums in the slurry. This could occur similarly to seawater, where calcium carbonate (CaCO_3_) minerals precipitate during freezing (Marion, 2001).

**FIGURE 4:**
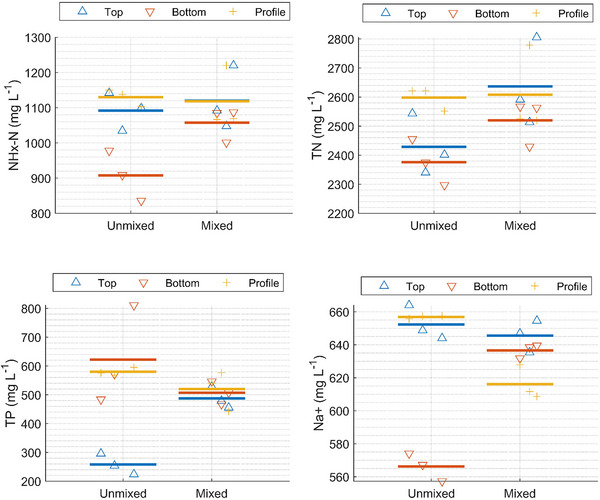
Total ammoniacal nitrogen (NHx‐N) (top left), total nitrogen (TN) (top right), total phosphorus (TP) (bottom left), and Na+ (bottom right) concentration for samples collected at the top, bottom, or as a profile from an unmixed or mixed liquid animal manure (slurry) tank. The symbols show the average of the subsamples, and the horizontal line shows the average values.

Clear differences between top and bottom samples were observed for several parameters, especially for the unmixed samples (Table 1; Figure 4). Bottom samples from the unmixed tank had higher DM and VS contents than top and profile samples, indicating sedimentation effects (Table 1; Figure S3). This pattern was further supported by the larger difference in DM between samples from mixed and unmixed tanks when sampling at the bottom (Table 1). Similar trends were observed for TP, with the highest concentrations found in the bottom samples from the unmixed tank (Table 1; Figure 4). The most pronounced differences in the top and bottom samples of the unmixed slurry were observed for DM and TP, with differences of approximately 80%–100% (Table 1). This agrees well with the findings by Higgins et al. (2004), who found linear correlations for nitrogen and phosphorus content with solids content. The unmixed slurry tank only had a thin crust, which was removed before sampling from the top as described in Section 2.2. The results and observations described above are likely to differ for slurry types; for example, for slurry with a thick crust where a high DM content in the top layer is present, thus the results might not be applicable for all types of slurry.

**TABLE 1:** Relative differences between top and bottom samples from mixed tank (M) and unmixed tank (U) (rows 1–2), between M and U samples for samples collected at top, profile, or bottom respectively (rows 3–5), and between top/bottom and profile samples for M (rows 6–7).

		NHx‐N	TN	TP	Na^+^	pH	pH^a^	DM	VS
M top	M bottom	5.7%	4.5%	1.4%	1.4%	−3.9%	−2.5%	−0.9%	0.6%
U top	U bottom	18.5%	2.2%	−82.8%	14.1%	2.8%	0.7%	−99.1%	−11.5%
M top	U top	2.5%	8.2%	61.6%	−1.0%	−0.8%	−0.5%	47.6%	11.6%
M profile	U profile	−1.0%	0.4%	−10.9%	−6.4%	1.5%	0.0%	−2.1%	−2.5%
M bottom	U bottom	15.3%	5.9%	−20.4%	11.7%	3.0%	2.7%	−57.6%	−0.5%
M top	M profile	−0.1%	−1.1%	6.4%	−4.7%	1.1%	1.8%	−1.5%	−1.1%
M bottom	M profile	−5.6%	−3.4%	−2.5%	3.3%	−0.1%	0.7%	2.4%	0.4%

*Note*: All samples were analyzed in the laboratory on the same day as collected. Total ammoniacal nitrogen (NHx‐N), total nitrogen (TN), total phosphorous (TP), sodium ions (Na^+^), pH, dry matter (DM), and volatile solids (VS). Negative values indicate that the second is smaller than the first.

^a^ Samples analyzed immediately after sampling at the tank.

Mixing the tank prior to sampling led to a more uniform distribution of DM, VS, and nutrients across all sampling points (Table 1; Figure 4). For instance, the difference in DM between top and bottom samples was reduced from 99% before mixing to 1% after mixing (Table 1). Consequently, the sampling method had a significant impact on the observed differences. After mixing, the variation between top and bottom samples was <6% for all parameters (Table 1), confirming that the sampling position becomes less critical when the slurry is thoroughly mixed. This observation is consistent with the findings of Johannesson et al. (2018).

Top sampling in unmixed tanks tended to underestimate parameters related to solids content, whereas bottom sampling tended to overestimate DM, VS, and TP due to sedimentation (Figure 4; Figures S3 and S8). Profile sampling provided a more balanced representation of the tank contents, especially in unmixed tanks, where the results from profile samples were similar to those obtained from the mixed tank. The more soluble compounds are more homogeneously distributed in the slurry. This is observed for parameters such as NHx‐N and Na^+^, top samples from the unmixed tank were also comparable to those from the mixed tank. These findings highlight the value of profile sampling as a practical alternative to full tank mixing when uniform sampling is required. Profile sampling might not be applicable to all types of slurry. It might be difficult to sample a representative part of a thick crust, especially if the crust material is coarse, for example, with large straw.

Sampling consistency by sampling three times at the same place and depth in a slurry tank was compared to tank variability by sampling from five locations around the edge of the tank at the same depth by Johannesson et al. (2018). The results from that study show low CV values for both tank variability and consistency. However, the CV for consistency sampling was higher than for tank variability in some cases, for example, 4.4% versus 1.4% for DM and 0.4% versus 0.1% for pH (Johannesson et al., 2018). Aguirre‐Villegas et al. (2018) combine sampling and modeling to conclude that a variation of 20%–30% can be obtained using three replicate samples for NHx‐N and TN and nine replicate samples for TP. The authors also concluded that estimates are improved by increasing the number of samples, but the improvement is not linear and may not be justifiable beyond a certain point (Aguirre‐Villegas et al., 2018). We note that if the replicate samples are unbiased (representative random sampling), the statistical deviation is reduced by a factor 1/sqrt(N), with N being the number of replicate samples [equivalent to the standard error decreasing by 1/sqrt(N)], meaning that doubling the number of samples will decrease the statistical deviation by 29%. Another study reported CVs for replicate samplings from an agitated slurry tank to 3%–7% for TN, 6%–8% for NHx‐N, and 7%–15% for TP, while the CVs were higher (13%–30% for all of TN, NHx‐N, and TP) for non‐agitated tanks (Dou et al., 2001, Table 2).

In the present study, three replicate samples were analyzed for each position (top, profile, and bottom) before and after mixing (Table S2). CVs for the mixed slurry tank ranged from 3% to 6% for TN, 5% to 8% for NHx‐N, and 8% to 13% for TP, which is comparable to the results by Dou et al. (2001). For the unmixed tank, CVs were 2%–4% for TN, 2%–8% for NHx‐N, and 2%–27% for TP, also comparable to the mixed tank in this study and lower than reported by Dou et al. (2001) for non‐agitated tanks. This indicates that sampling from an unmixed tank can result in high precision but insufficient accuracy.

Importantly, for one of three replicate samples sampled at the bottom of the unmixed tank, the results show a clearly higher DM content compared to the other two (roughly 9.3% vs. 7.4%, also in multiple samples analyzed upon storage; see Figure S3). This is also reflected by high TP for this replicate sample (810 mg L^−1^ for this sample vs. 483 and 571 mg L^−1^ for the other two of the three replicate samples; see Figure 4; Figure S6). Similar is observed for the top samples from the unmixed tank, whereas results of the profile samples show much less deviation (see Figure 4; Table S6; Figures S3–S8). These observations demonstrate the difficulty of representative sampling and the importance of taking multiple replicate samples from the slurry tank of interest.

**FIGURE 5:**
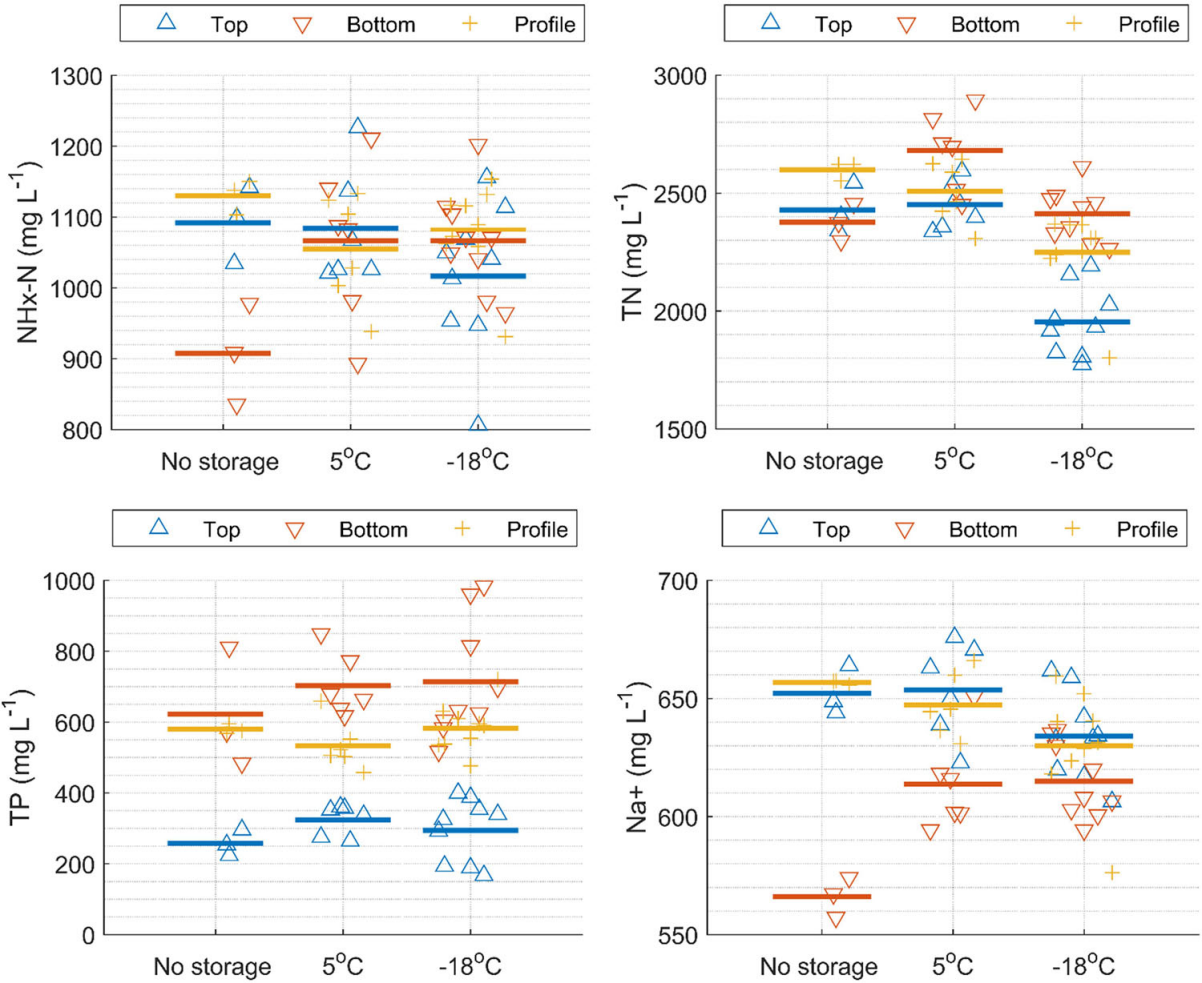
Total ammoniacal nitrogen (NHx‐N) (top left), total nitrogen (TN) (top right), total phosphorus (TP) (bottom left), and Na^+^ (bottom right) concentration for samples collected at the top, bottom, or as a profile from an unmixed liquid animal manure (slurry) tank and analyzed immediately after collection or stored either at 5°C or ‐18°C. The symbols show the average of the subsamples, and the horizontal line shows the average values.

The results of the NHx‐N, TN, TP, Na^+^, and DM measurements show little effect of sample storage, and for most of the profile samples the results are relatively stable upon storage at 5°C (1 and 4 weeks) and −18°C (4, 12, and 24 weeks) (Figure 5; Figure S3). Note that, for example, the highest TP results from bottom samplings originate from the same replicate sample as mentioned above. This occurs as the storage tests were performed on samples from the unmixed tank, which show large variations between the replicate samples (particularly for top and bottom samples, respectively). However, for the samples taken from the bottom of the tank, with high DM and high inhomogeneity, the NHx‐N and Na^+^ results appear higher upon storage (16.1% for NHx‐N and 8.1% for Na, given as mean increases for bottom samples analyzed after storage (at 5°C) versus analysis directly after sampling). This may be due to the release of NH_4_^+^ and Na^+^ ions from increased mineralization during the thawing process.

On the other hand, for the top samples, TN is lower after freezing and thawing compared to the results of the fresh and refrigerated samples. Compared to samples stored at 5°C, storage at −18°C gave an average of 20% lower TN and, on average, 19% and 13% lower TN than bottom and profile samples stored at −18°C, respectively. This may possibly occur due to increased mineralization of organic nitrogen during the thawing process (to NH_4_^+^) followed by loss of NHx‐N through NH_3_ volatilization due to the high pH (7.8–8.2, see Figure 3) or other N volatilization (e.g., from nitrification‐denitrification processes). Increased mineralization, volatilization, and several other effects upon freezing have in some cases previously been observed for soils (DeLuca et al., 1992; Herrmann & Witter, 2002; Liu et al., 2024), and freeze‐thaw cycles have been shown to change physiochemical properties of pig manure (Chen et al., 2019). In our study, mineralization would agree with the slightly increased NHx‐N content for the bottom samples upon freezing (see above and Figure 5), and the lower TN for the top samples agrees with higher pH for these samples (Figure 3). Another explanation may relate to the combination of low DM content and elevated pH for the top samples, which may cause a slight effect on the measured organic‐nitrogen fraction of TN.

In summary, this study highlights several practical insights for slurry sampling and storage. Profile sampling provided the most representative results in unmixed tanks and can serve as a practical alternative when tank mixing is not feasible. Top sampling tended to underestimate solids and nutrient contents, while bottom sampling tended to overestimate them due to sedimentation. Thorough mixing minimized biases but is time and energy consuming and may increase gaseous emissions. Storage at 5°C preserved sample integrity for several weeks, whereas freezing at −18°C caused systematic changes in pH. These findings provide guidance for both practitioners and researchers on how to design robust strategies for slurry sampling and sample storage before analysis.

These results partly support our hypotheses. Hypothesis (i) was confirmed as profile samples from an unmixed tank provided results comparable to a mixed tank. Hypothesis (ii) was also supported, since the sampling method strongly affected NHx‐N, TN, TP, and DM, but only minor effects on pH were observed. Hypothesis (iii) was not fully supported because while storage in a refrigerator preserved sample integrity, freezing altered pH.

Finally, we note that while this study involved slurry samples with different properties and nutrient contents (due to different samplings), the samples were all aliquots of slurry from the same slurry tank. Thereby, the results do not provide a statistical overview of the observed effects, but the study exemplifies effects of sampling and storage. The parameters analyzed were limited to NHx‐N, TN, TP, Na^+^, DM, VS, and pH; thus, the results might not be applicable to other analytes such as inorganic trace elements or organic pollutants.

## 4 CONCLUSION

This study demonstrates slurry sampling methods and storage conditions can impact the chemical properties of slurry. Mixing the slurry tank for 4 h resulted in uniform samples across different depths, indicating complete mixing. Profile sampling from an unmixed tank provided representative samples comparable to a thoroughly mixed tank, offering an alternative to mixing the entire tank. Sedimentation was evident in unmixed slurry, with higher DM and VS at the bottom compared to the top. The pH of the slurry changed after sampling and should be monitored closely depending on the purpose of the measurement. No change in pH was observed between measurements in the laboratory within a few hours after sampling compared to storage in a refrigerator for 1 or 4 weeks. However, the pH increased significantly after storage in a freezer for 4–24 weeks compared to storage in a refrigerator for 1–4 weeks. These findings underscore the importance of standardized sampling and storage protocols to ensure accurate slurry analysis for nutrient management. Implementing such protocols will enhance the reliability of nutrient measurements. Future research should verify these results for other types of tanks and types of slurry. Furthermore, optimizing sampling and storage techniques to further improve the accuracy of slurry analysis is important.

## AUTHOR CONTRIBUTIONS

**Jesper Nørlem Kamp**: Conceptualization; data curation; formal analysis; funding acquisition; investigation; methodology; project administration; validation; visualization; writing—original draft; writing—review and editing. **Morten Kjærulff Sørensen**: Investigation; methodology; validation; visualization; writing—original draft; writing—review and editing. **Johanna Pedersen**: Conceptualization; funding acquisition; investigation; methodology; validation; visualization; writing—original draft; writing—review and editing.

## CONFLICT OF INTEREST STATEMENT

Morten Kjærulff Sørensen has affiliation to NanoNord A/S selling the applied Tveskaeg NMR sensor. The remaining authors declare no conflicts of interest.

## Supporting information



The supplementary materials provide a complete overview of all analyzed samples, including a detailed table on effect of mixing. In addition, supplementary figures and photographs of the slurry tank and the sampling device are included to support the methodology and sampling approach.

## Data Availability

Calculations and data can be found in a GitHub repository (https://github.com/AU‐BCE‐EE/Kamp‐2025‐Slurry‐Sampling).
