# Supplementary material for: Full‐scale slurry tank sampling: Effects of sampling strategy and sample storage on measured physical and chemical properties
Source: J Environ Qual. 2025 Dec 22;55(1):e70128. doi: 10.1002/jeq2.70128 (PMC12723325; doi:10.1002/jeq2.70128)
Supplement: Supplementary file 1 — The supplementary materials provide a complete overview of all analyzed samples, including a detailed table on effect of mixing. In addition, supplementary figures and photographs of the slurry tank and the sampling device are included to support the methodology and sampling approach. [file JEQ2-55-0-s001.docx]

# Supporting information

# Full-scale slurry tank sampling: Effects of sampling strategy and sample storage on measured physical and chemical properties

Jesper Nørlem Kamp^1,^*, Morten Kjærulff Sørensen^1,2^, Johanna Pedersen^1^

^1^ Department of Biological and Chemical Engineering, Aarhus University, Aarhus, Denmark

^2^ NanoNord A/S, Aalborg, Denmark

Table S1. Overview of sampling and storage conditions along with analyses conducted for each sample.

| ID | Name | Storage | Sampling | Analysis | | | | |
| --- | --- | --- | --- | --- | --- | --- | --- | --- |
|  |  |  |  |  | + | * | * | * |
| 1 | U-T-1 | Fresh (N) | Unmixed, top, 1 | NMR | 4 | DM | VS | pH |
| 2 | U-T-1 | Freezer 1 month (F-4) | Unmixed, top, 1 | NMR | 3 | DM |  | pH |
| 3 | U-T-1 | Freezer 3 month (F-12) | Unmixed, top, 1 | NMR | 3 | DM |  | pH |
| 4 | U-T-1 | Refrigerator 1 week (R-1) | Unmixed, top, 1 | NMR | 3 | DM |  | pH |
| 5 | U-T-1 | Refrigerator 1 month (R-4) | Unmixed, top, 1 | NMR | 3 | DM |  | pH |
| 6 | U-T-1 | Freezer 6 month (F-24) | Unmixed, top, 1 | NMR | 3 | DM |  | pH |
| 7 | U-T-2 | Fresh (N) | Unmixed, top, 2 | NMR | 4 | DM | VS | pH |
| 8 | U-T-2 | Freezer 1 month (F-4) | Unmixed, top, 2 | NMR | 3 | DM |  | pH |
| 9 | U-T-2 | Freezer 3 month (F-12) | Unmixed, top, 2 | NMR | 3 | DM |  | pH |
| 10 | U-T-2 | Refrigerator 1 week (R-1) | Unmixed, top, 2 | NMR | 3 | DM |  | pH |
| 11 | U-T-2 | Refrigerator 1 month (R-4) | Unmixed, top, 2 | NMR | 3 | DM |  | pH |
| 12 | U-T-2 | Freezer 6 month (F-24) | Unmixed, top, 2 | NMR | 3 | DM |  | pH |
| 13 | U-T-3 | Fresh (N) | Unmixed, top, 3 | NMR | 4 | DM | VS | pH |
| 14 | U-T-3 | Freezer 1 month (F-4) | Unmixed, top, 3 | NMR | 2 | DM |  | pH |
| 15 | U-T-3 | Freezer 3 month (F-12) | Unmixed, top, 3 | NMR | 2 | DM |  | pH |
| 16 | U-T-3 | Refrigerator 1 week (R-1) | Unmixed, top, 3 | NMR | 2 | DM |  | pH |
| 17 | U-T-3 | Refrigerator 1 month (R-4) | Unmixed, top, 3 | NMR | 2 | DM |  | pH |
| 18 | U-T-3 | Freezer 6 month (F-24) | Unmixed, top, 3 | NMR | 2 | DM |  | pH |
| 19 | U-B-1 | Fresh (N) | Unmixed, bottom, 1 | NMR | 4 | DM | VS | pH |
| 20 | U-B-1 | Freezer 1 month (F-4) | Unmixed, bottom, 1 | NMR | 3 | DM |  | pH |
| 21 | U-B-1 | Freezer 3 month (F-12) | Unmixed, bottom, 1 | NMR | 3 | DM |  | pH |
| 22 | U-B-1 | Refrigerator 1 week (R-1) | Unmixed, bottom, 1 | NMR | 3 | DM |  | pH |
| 23 | U-B-1 | Refrigerator 1 month (R-4) | Unmixed, bottom, 1 | NMR | 3 | DM |  | pH |
| 24 | U-B-1 | Freezer 6 month (F-24) | Unmixed, bottom, 1 | NMR | 3 | DM |  | pH |
| 25 | U-B-2 | Fresh (N) | Unmixed, bottom, 2 | NMR | 4 | DM | VS | pH |
| 26 | U-B-2 | Freezer 1 month (F-4) | Unmixed, bottom, 2 | NMR | 3 | DM |  | pH |
| 27 | U-B-2 | Freezer 3 month (F-12) | Unmixed, bottom, 2 | NMR | 3 | DM |  | pH |
| 28 | U-B-2 | Refrigerator 1 week (R-1) | Unmixed, bottom, 2 | NMR | 3 | DM |  | pH |
| 29 | U-B-2 | Refrigerator 1 month (R-4) | Unmixed, bottom, 2 | NMR | 3 | DM |  | pH |
| 30 | U-B-2 | Freezer 6 month (F-24) | Unmixed, bottom, 2 | NMR | 3 | DM |  | pH |
| 31 | U-B-3 | Fresh (N) | Unmixed, bottom, 3 | NMR | 4 | DM | VS | pH |
| 32 | U-B-3 | Freezer 1 month (F-4) | Unmixed, bottom, 3 | NMR | 2 | DM |  | pH |
| 33 | U-B-3 | Freezer 3 month (F-12) | Unmixed, bottom, 3 | NMR | 2 | DM |  | pH |
| 34 | U-B-3 | Refrigerator 1 week (R-1) | Unmixed, bottom, 3 | NMR | 2 | DM |  | pH |
| 35 | U-B-3 | Refrigerator 1 month (R-4) | Unmixed, bottom, 3 | NMR | 2 | DM |  | pH |
| 36 | U-B-3 | Freezer 6 month (F-24) | Unmixed, bottom, 3 | NMR | 2 | DM |  | pH |
| 37 | U-P-1 | Fresh (N) | Unmixed, profile, 1 | NMR | 4 | DM | VS | pH |
| 38 | U-P-1 | Freezer 1 month (F-4) | Unmixed, profile, 1 | NMR | 3 | DM |  | pH |
| 39 | U-P-1 | Freezer 3 month (F-12) | Unmixed, profile, 1 | NMR | 3 | DM |  | pH |
| 40 | U-P-1 | Refrigerator 1 week (R-1) | Unmixed, profile, 1 | NMR | 3 | DM |  | pH |
| 41 | U-P-1 | Refrigerator 1 month (R-4) | Unmixed, profile, 1 | NMR | 3 | DM |  | pH |
| 42 | U-P-1 | Freezer 6 month (F-24) | Unmixed, profile, 1 | NMR | 3 | DM |  | pH |
| 43 | U-P-2 | Fresh (N) | Unmixed, profile, 2 | NMR | 4 | DM | VS | pH |
| 44 | U-P-2 | Freezer 1 month (F-4) | Unmixed, profile, 2 | NMR | 3 | DM |  | pH |
| 45 | U-P-2 | Freezer 3 month (F-12) | Unmixed, profile, 2 | NMR | 3 | DM |  | pH |
| 46 | U-P-2 | Refrigerator 1 week (R-1) | Unmixed, profile, 2 | NMR | 3 | DM |  | pH |
| 47 | U-P-2 | Refrigerator 1 month (R-4) | Unmixed, profile, 2 | NMR | 3 | DM |  | pH |
| 48 | U-P-2 | Freezer 6 month (F-24) | Unmixed, profile, 2 | NMR | 3 | DM |  | pH |
| 49 | U-P-3 | Fresh (N) | Unmixed, profile, 3 | NMR | 4 | DM | VS | pH |
| 50 | U-P-3 | Freezer 1 month (F-4) | Unmixed, profile, 3 | NMR | 2 | DM |  | pH |
| 51 | U-P-3 | Freezer 3 month (F-12) | Unmixed, profile, 3 | NMR | 2 | DM |  | pH |
| 52 | U-P-3 | Refrigerator 1 week (R-1) | Unmixed, profile, 3 | NMR | 2 | DM |  | pH |
| 53 | U-P-3 | Refrigerator 1 month (R-4) | Unmixed, profile, 3 | NMR | 2 | DM |  | pH |
| 54 | U-P-3 | Freezer 6 month (F-24) | Unmixed, profile, 3 | NMR | 2 | DM |  | pH |
| 55 | M-T-1 | Fresh (N) | Mixed, top, 1 | NMR | 3 | DM | VS | pH |
| 56 | M-T-2 | Fresh (N) | Mixed, top, 2 | NMR | 3 | DM |  | pH |
| 57 | M-T-3 | Fresh (N) | Mixed, top, 3 | NMR | 2 | DM |  | pH |
| 58 | M-B-1 | Fresh (N) | Mixed, bottom, 1 | NMR | 3 | DM | VS | pH |
| 59 | M-B-2 | Fresh (N) | Mixed, bottom, 2 | NMR | 3 | DM |  | pH |
| 60 | M-B-3 | Fresh (N) | Mixed, bottom, 3 | NMR | 2 | DM |  | pH |
| 61 | M-P-1 | Fresh (N) | Mixed, profile, 1 | NMR | 3 | DM | VS | pH |
| 62 | M-P-2 | Fresh (N) | Mixed, profile, 2 | NMR | 3 | DM |  | pH |
| 63 | M-P-3 | Fresh (N) | Mixed, profile, 3 | NMR | 2 | DM |  | pH |

+ Number of subsamples for NMR measurements.

*Duplicate samples

Table S2. Mixed (M) or unmixed (U) slurry samples taken as bottom (B), profile (P), or top (T) sample and analyzed the same day as collected. Overview of summarized values for number of samples (N), average (avg.), standard deviation (SD), minimum value (min), maximum value (max), and coefficient of variation (CV). Each sample (N) represents several subsamples, see Table S3.

| NH_x_-N | | | | | | | |
| --- | --- | --- | --- | --- | --- | --- | --- |
| Mixing | Position | N | Avg (mg L^-1^) | SD (mg L^-1^) | Min (mg L^-1^) | Max (mg L^-1^) | CV (%) |
| M | B | 3 | 1057.7 | 49.0 | 1001.1 | 1086.9 | 4.6% |
| M | P | 3 | 1118.6 | 87.8 | 1066.7 | 1220.0 | 7.8% |
| M | T | 3 | 1119.9 | 89.7 | 1047.7 | 1220.3 | 8.0% |
| U | B | 3 | 907.4 | 71.1 | 835.6 | 977.9 | 7.8% |
| U | P | 3 | 1130.4 | 24.4 | 1103.2 | 1150.2 | 2.2% |
| U | T | 3 | 1091.8 | 54.1 | 1034.5 | 1142.0 | 5.0% |
| TN | | | | | | | |
| Mixing | Position | N | Avg (µg m^-3^) | SD (µg m^-3^) | Min (µg m^-3^) | Max (µg m^-3^) | CV (%) |
| M | B | 3 | 2520.1 | 78.4 | 2429.5 | 2567.3 | 3.1% |
| M | P | 3 | 2607.8 | 147.7 | 2520.0 | 2778.3 | 5.7% |
| M | T | 3 | 2636.8 | 151.2 | 2513.5 | 2805.4 | 5.7% |
| U | B | 3 | 2375.7 | 79.2 | 2297.0 | 2455.4 | 3.3% |
| U | P | 3 | 2598.1 | 40.1 | 2551.8 | 2621.3 | 1.5% |
| U | T | 3 | 2428.5 | 104.1 | 2340.5 | 2543.4 | 4.3% |
| TP | | | | | | | |
| Mixing | Position | N | Avg (mg L^-1^) | SD (mg L^-1^) | Min (mg L^-1^) | Max (mg L^-1^) | CV (%) |
| M | B | 3 | 507.1 | 39.5 | 467.4 | 546.3 | 7.8% |
| M | P | 3 | 519.8 | 69.8 | 442.0 | 576.9 | 13.4% |
| M | T | 3 | 487.6 | 37.3 | 455.5 | 528.4 | 7.6% |
| U | B | 3 | 622.2 | 169.3 | 483.9 | 811.0 | 27.2% |
| U | P | 3 | 579.8 | 13.7 | 568.3 | 595.0 | 2.4% |
| U | T | 3 | 257.9 | 36.2 | 223.9 | 296.0 | 14.0% |
| Na^+^ | | | | | | | |
| Mixing | Position | N | Avg (mg L^-1^) | SD (mg L^-1^) | Min (mg L^-1^) | Max (mg L^-1^) | CV (%) |
| M | B | 3 | 636.6 | 4.2 | 631.8 | 639.5 | 0.7% |
| M | P | 3 | 616.1 | 10.3 | 608.8 | 627.8 | 1.7% |
| M | T | 3 | 645.6 | 9.7 | 635.4 | 654.6 | 1.5% |
| U | B | 3 | 566.2 | 8.4 | 557.3 | 574.1 | 1.5% |
| U | P | 3 | 656.8 | 1.0 | 655.7 | 657.5 | 0.2% |
| U | T | 3 | 652.2 | 10.4 | 644.0 | 664.0 | 1.6% |
| pH | | | | | | | |
| Mixing | Position | N | Avg | SD | Min | Max | CV (%) |
| M | B | 3 | 7.42 | 0.01 | 7.4 | 7.4 | 0.2% |
| M | P | 3 | 7.43 | 0.02 | 7.4 | 7.4 | 0.2% |
| M | T | 3 | 7.35 | 0.05 | 7.3 | 7.4 | 0.6% |
| U | B | 3 | 7.20 | 0.01 | 7.2 | 7.2 | 0.2% |
| U | P | 3 | 7.31 | 0.03 | 7.3 | 7.3 | 0.4% |
| U | T | 3 | 7.41 | 0.03 | 7.4 | 7.4 | 0.4% |
| M* | B | 3 | 7.33 | 0.02 | 7.3 | 7.4 | 0.3% |
| M* | P | 3 | 7.28 | 0.01 | 7.3 | 7.3 | 0.1% |
| M* | T | 3 | 7.15 | 0.02 | 7.1 | 7.2 | 0.2% |
| U* | B | 18 | 7.13 | 0.01 | 7.1 | 7.2 | 0.1% |
| U* | P | 18 | 7.28 | 0.02 | 7.3 | 7.3 | 0.2% |
| U* | T | 18 | 7.18 | 0.02 | 7.1 | 7.2 | 0.3% |
| DM | | | | | | | |
| Mixing | Position | N | Avg (%) | SD (%) | Min (%) | Max (%) | CV (%) |
| M | B | 3 | 4.5 | 0.0 | 4.4 | 4.5 | 0.6% |
| M | P | 3 | 4.4 | 0.1 | 4.3 | 4.4 | 1.3% |
| M | T | 3 | 4.4 | 0.0 | 4.4 | 4.4 | 0.6% |
| U | B | 3 | 8.1 | 1.2 | 7.2 | 9.4 | 14.6% |
| U | P | 3 | 4.4 | 0.1 | 4.4 | 4.5 | 1.3% |
| U | T | 3 | 2.7 | 0.3 | 2.5 | 3.1 | 11.5% |
| VS | | | | | | | |
| Mixing | Position | N | Avg. (%) | SD (%) | Min (%) | Max (%) | CV (%) |
| M | B | 3 | 74.6 | 0.1 | 74.5 | 74.7 | 0.1% |
| M | P | 3 | 74.3 | 0.4 | 73.9 | 74.7 | 0.5% |
| M | T | 3 | 75.1 | 0.1 | 74.9 | 75.1 | 0.1% |
| U | B | 3 | 75.0 | 10.8 | 62.6 | 81.4 | 14.3% |
| U | P | 3 | 76.2 | 0.2 | 75.9 | 76.3 | 0.3% |
| U | T | 3 | 66.8 | 2.8 | 65.0 | 70.0 | 4.2% |

* Samples analyzed immediately after sampling at the tank.


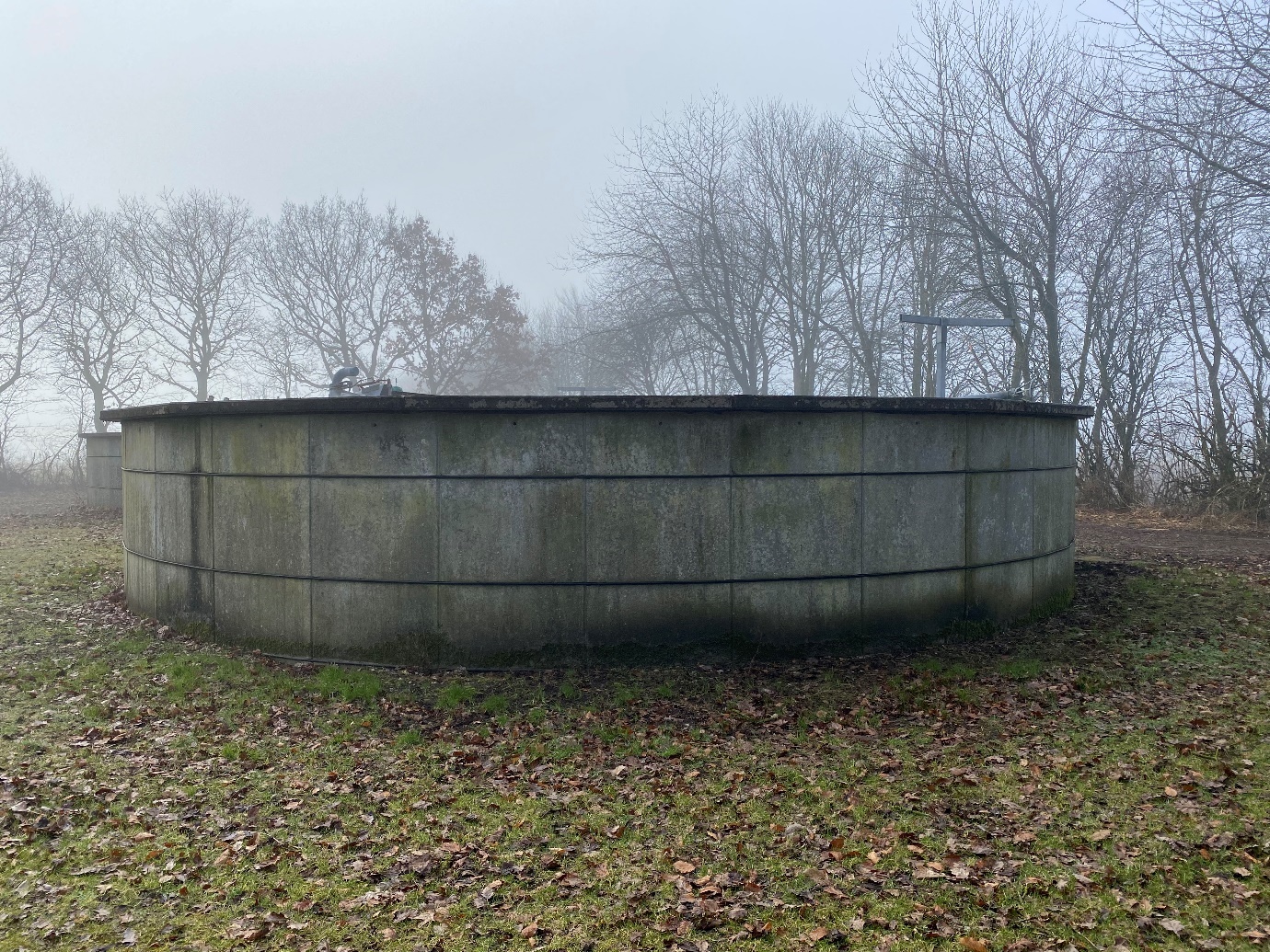


Figure S1. Concrete slurry tank where the slurry samples were collected.

| 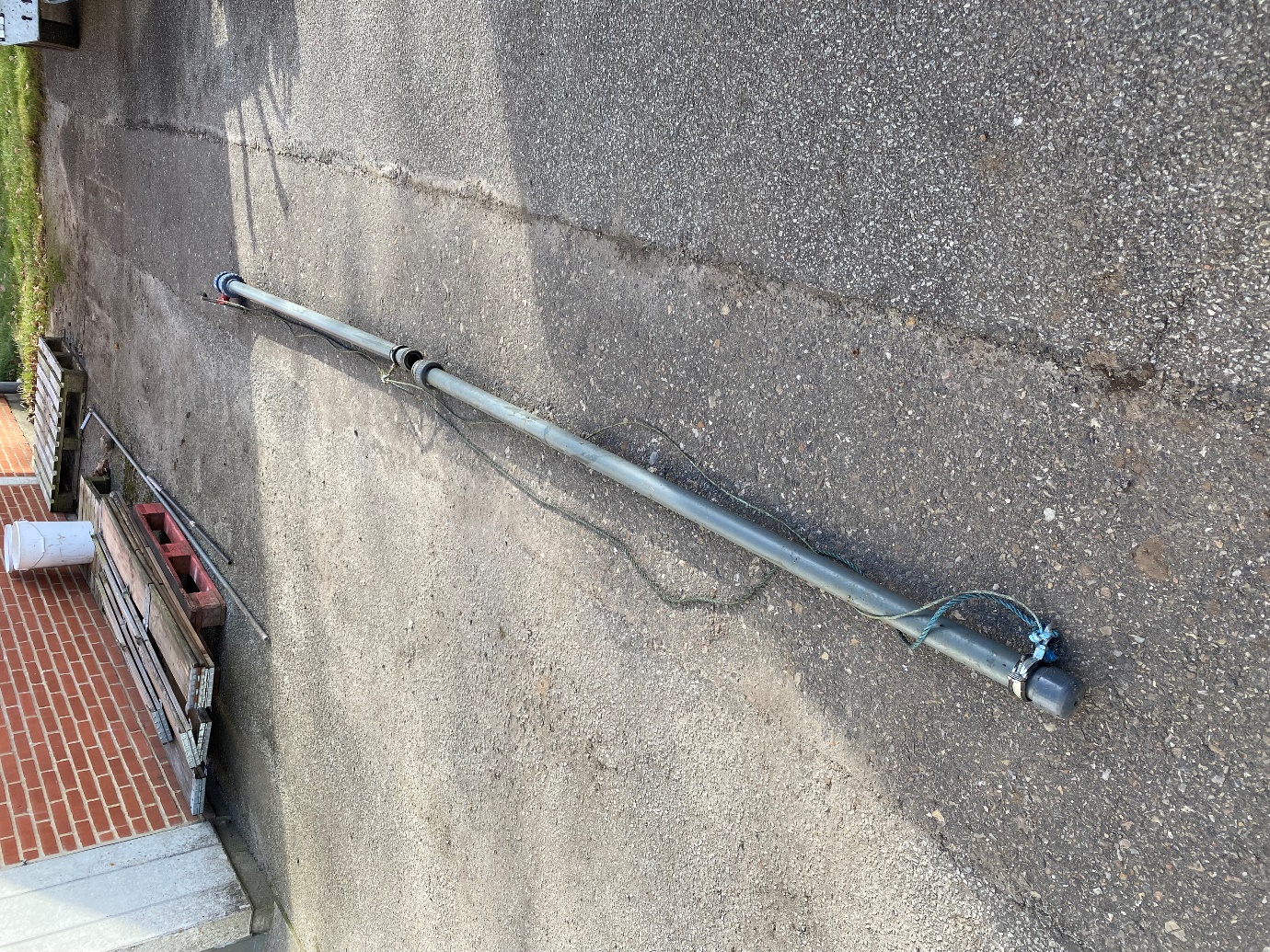 | 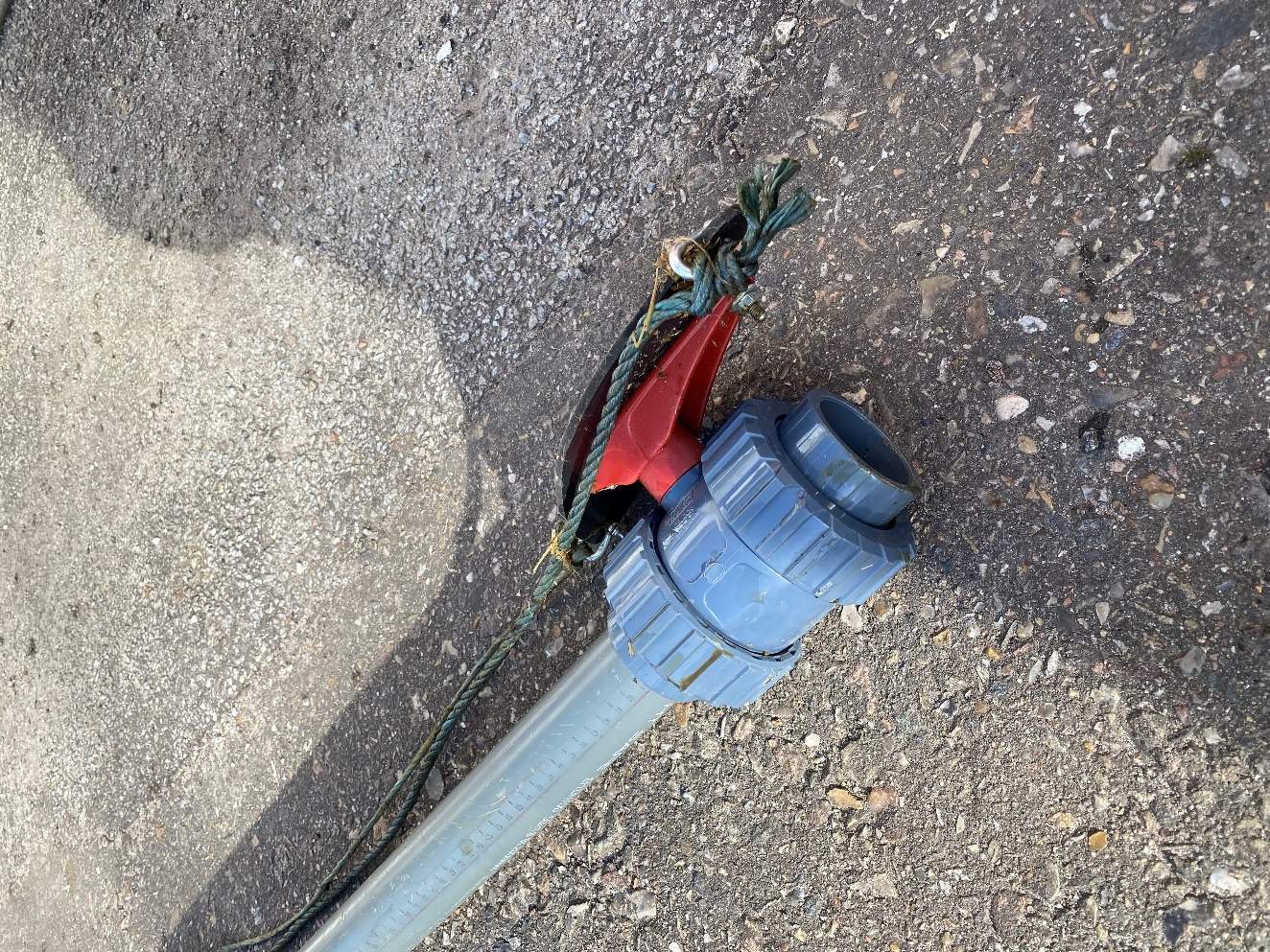 |
| --- | --- |

Figure S2. Sampling device for profile sampling.


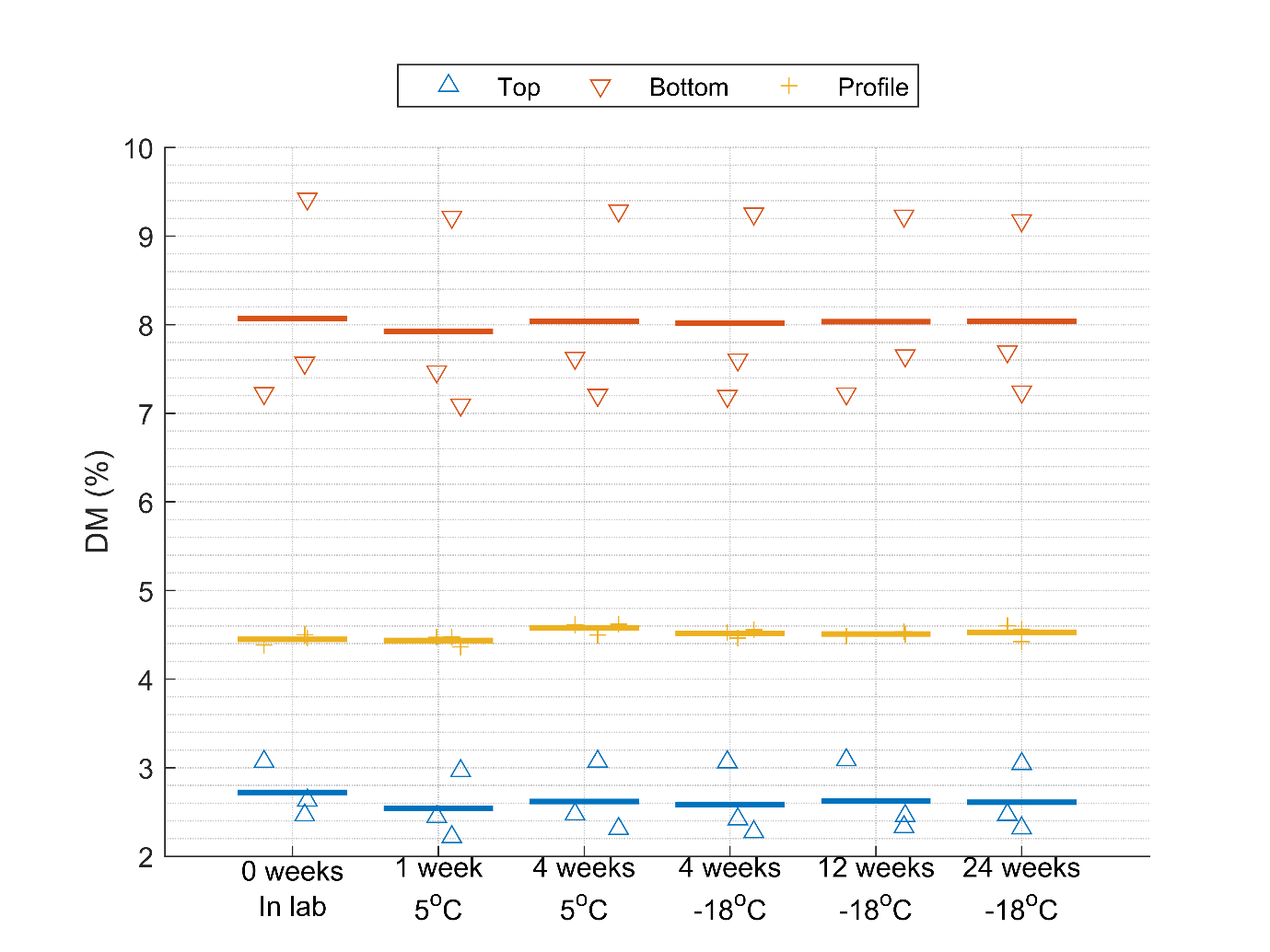


Figure S3. DM measurements in samples from an unmixed slurry tank. Measurements of samples directly after collection, after storage in the refrigerator (5°C) for 1 or 4 weeks, and after storage in a freezer (-18°C) for 4, 12, or 24 weeks. The symbols show individual measurements, and the horizontal line shows the average values.


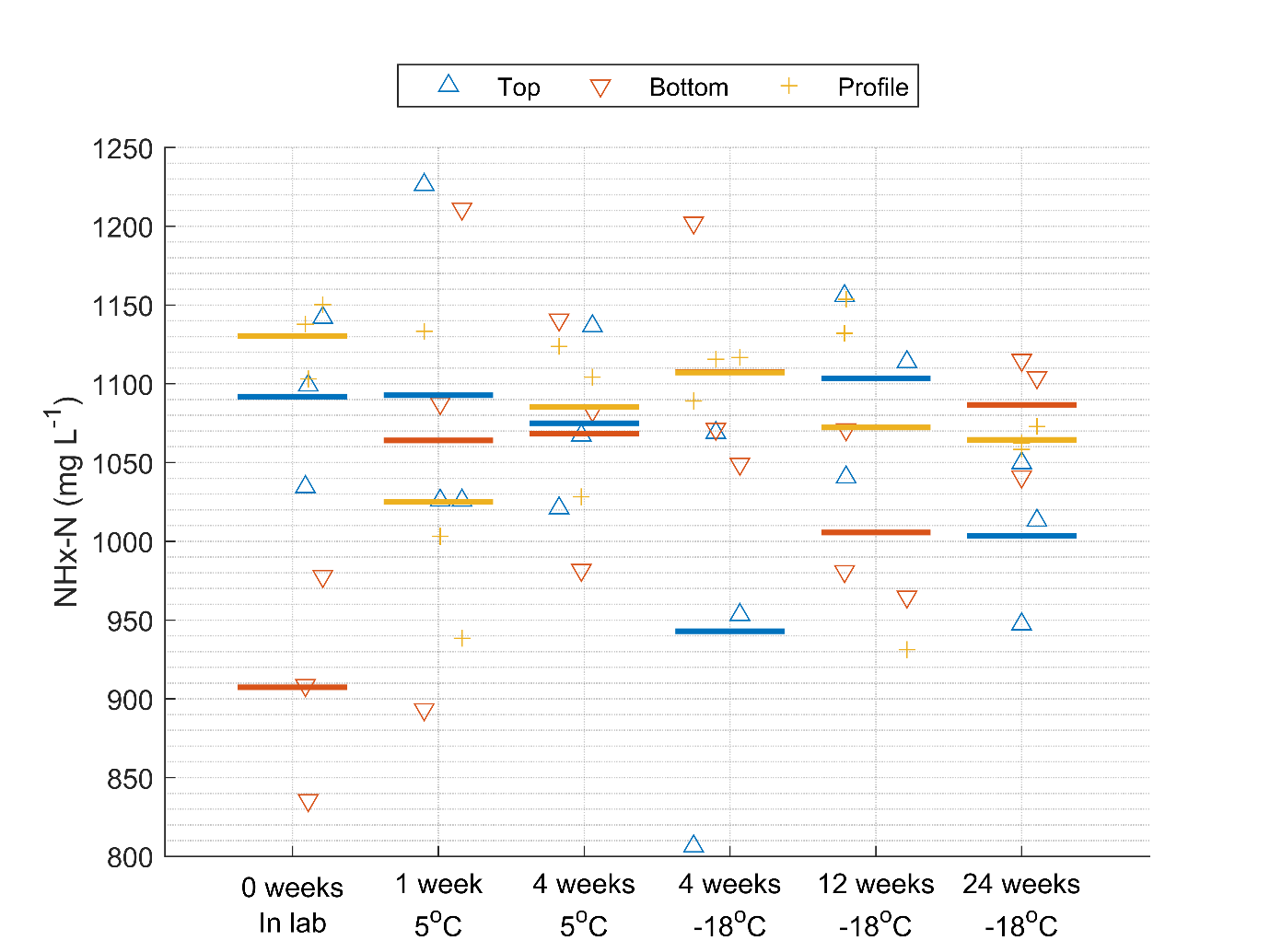


Figure S4. NHx-N concentration measurements in samples from an unmixed slurry tank. Measurements of samples directly after collection, after storage in the refrigerator (5°C) for 1 or 4 weeks, and after storage in a freezer (-18°C) for 4, 12, or 24 weeks. The symbols show individual measurements, and the horizontal line shows the average values.


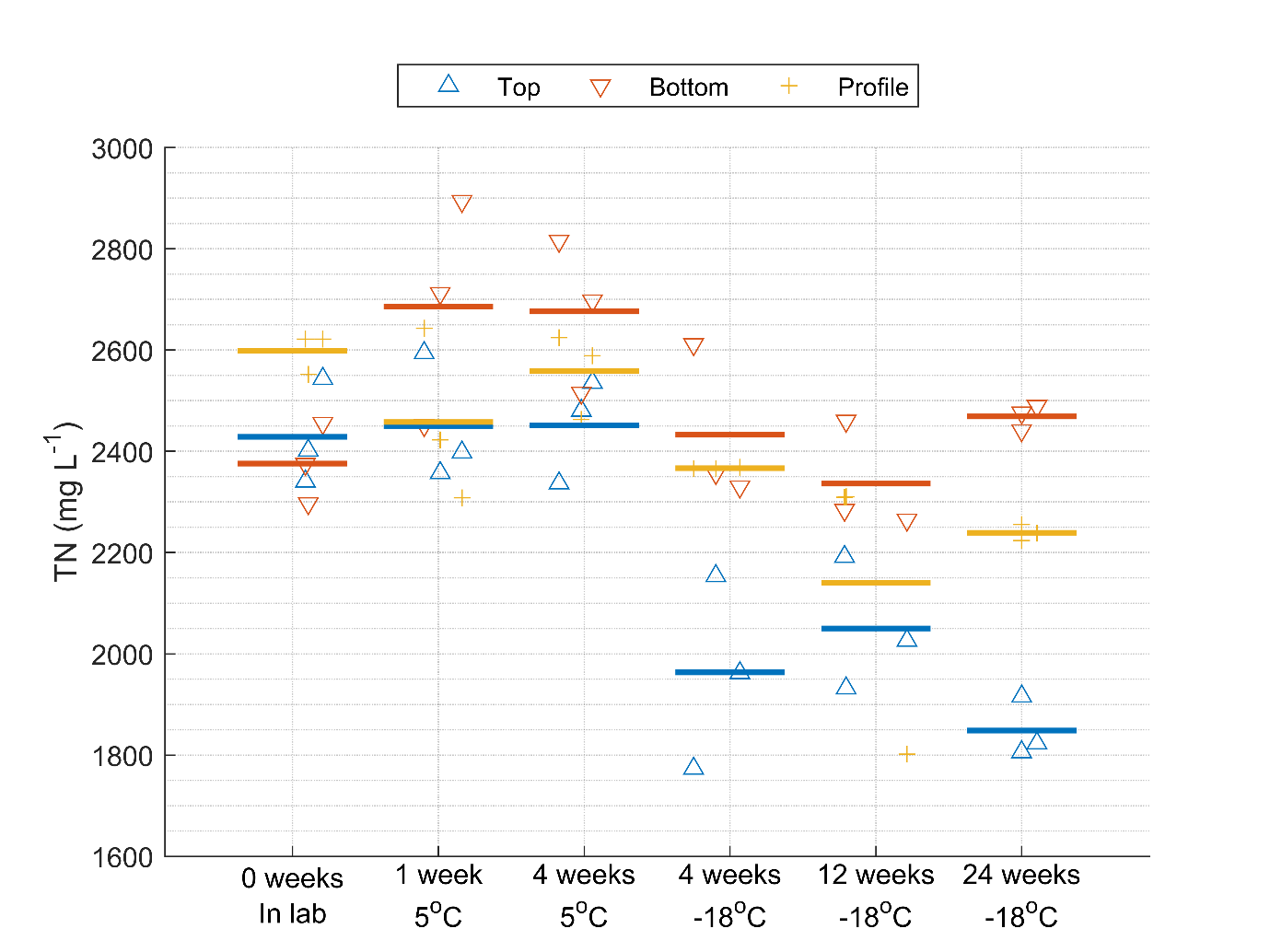


Figure S5. TN concentration measurements in samples from an unmixed slurry tank. Measurements of samples directly after collection, after storage in the refrigerator (5°C) for 1 or 4 weeks, and after storage in a freezer (-18°C) for 4, 12, or 24 weeks. The symbols show individual measurements, and the horizontal line shows the average values.


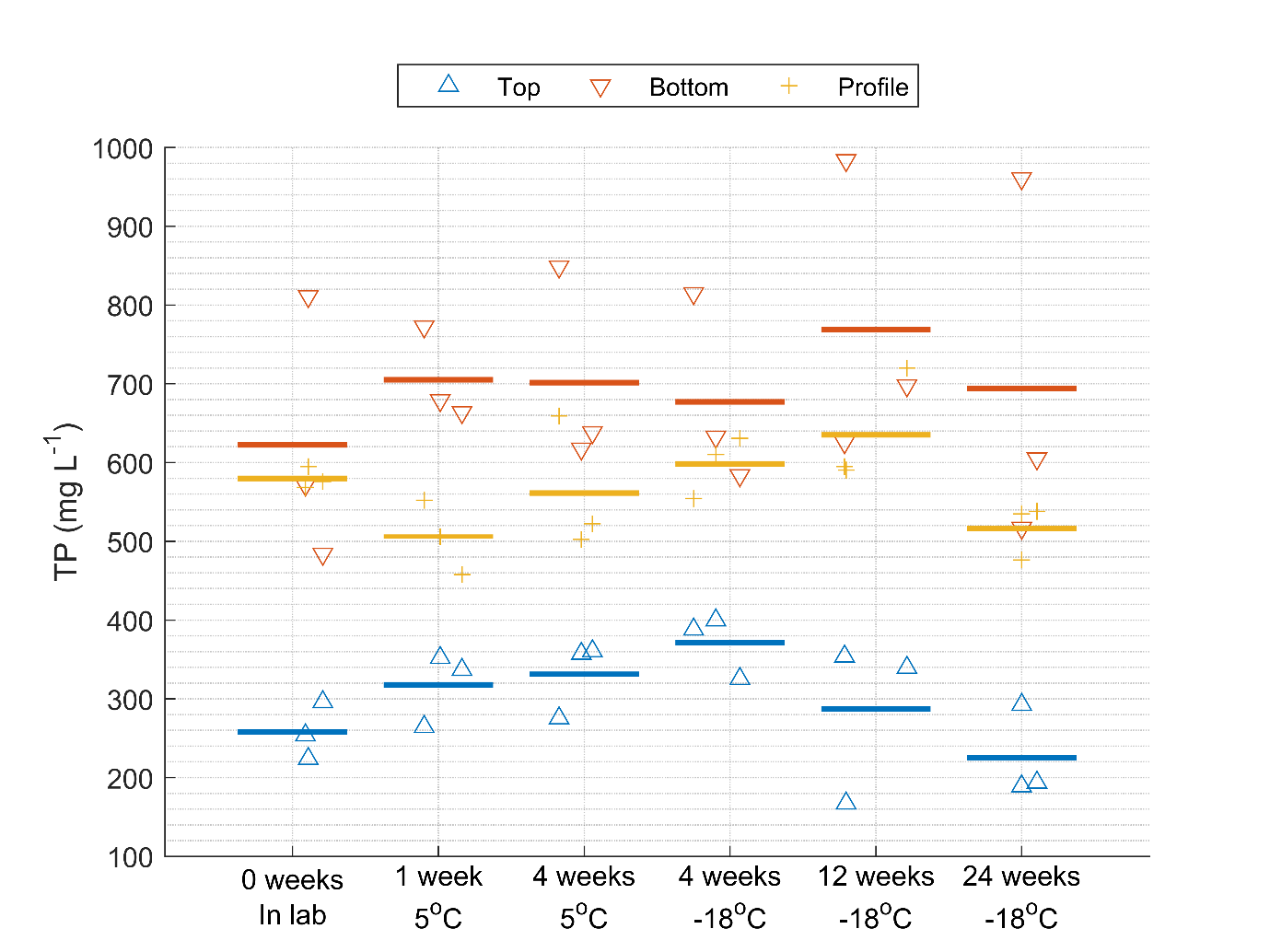


Figure S6. TP concentration measurements in samples from an unmixed slurry tank. Measurements of samples directly after collection, after storage in the refrigerator (5°C) for 1 or 4 weeks, and after storage in a freezer (-18°C) for 4, 12, or 24 weeks. The symbols show individual measurements, and the horizontal line shows the average values.


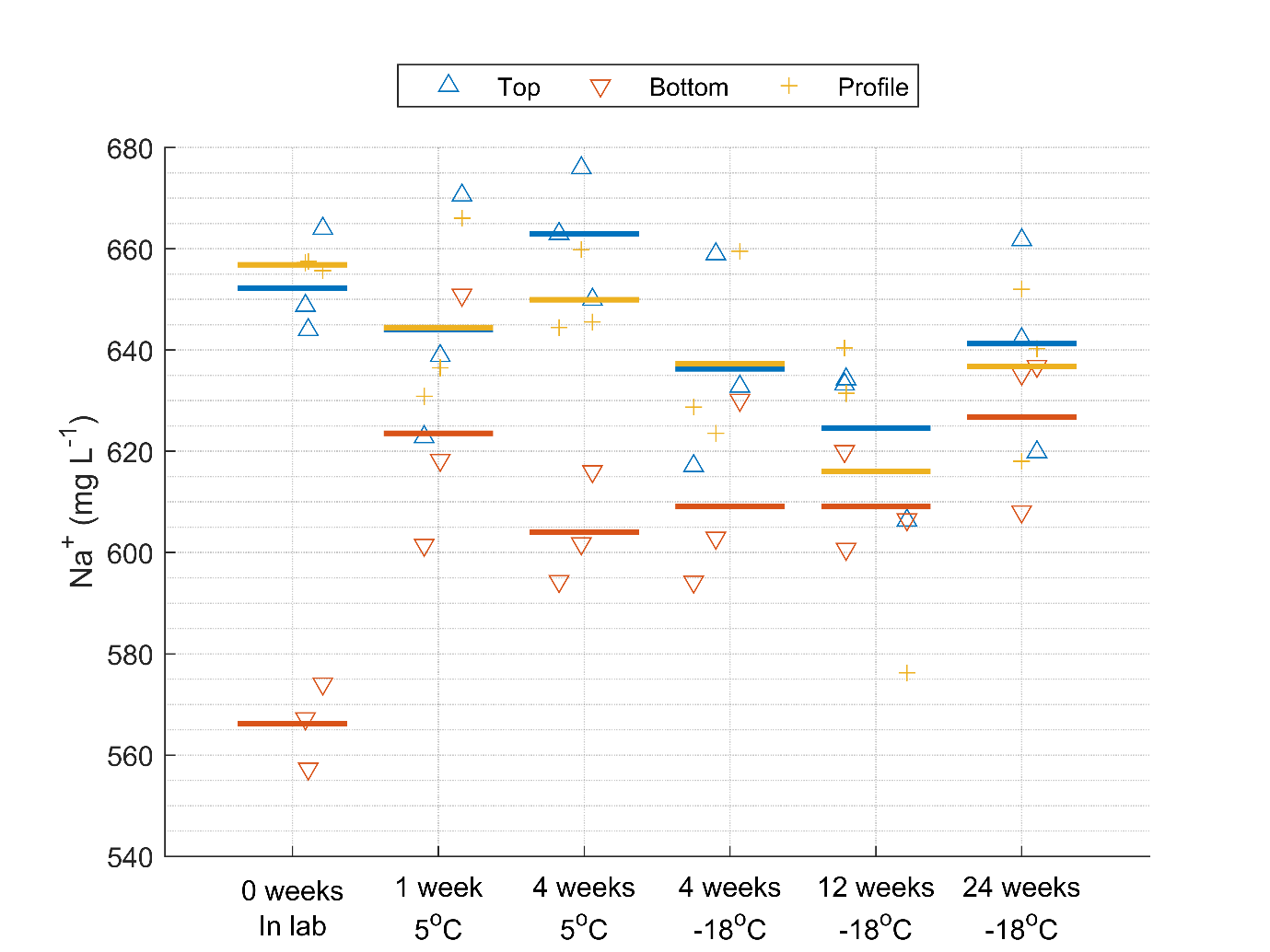


Figure S7. Na^+^ concentration measurements in samples from an unmixed slurry tank. Measurements of samples directly after collection, after storage in the refrigerator (5°C) for 1 or 4 weeks, and after storage in a freezer (-18°C) for 4, 12, or 24 weeks. The symbols show individual measurements, and the horizontal line shows the average values.


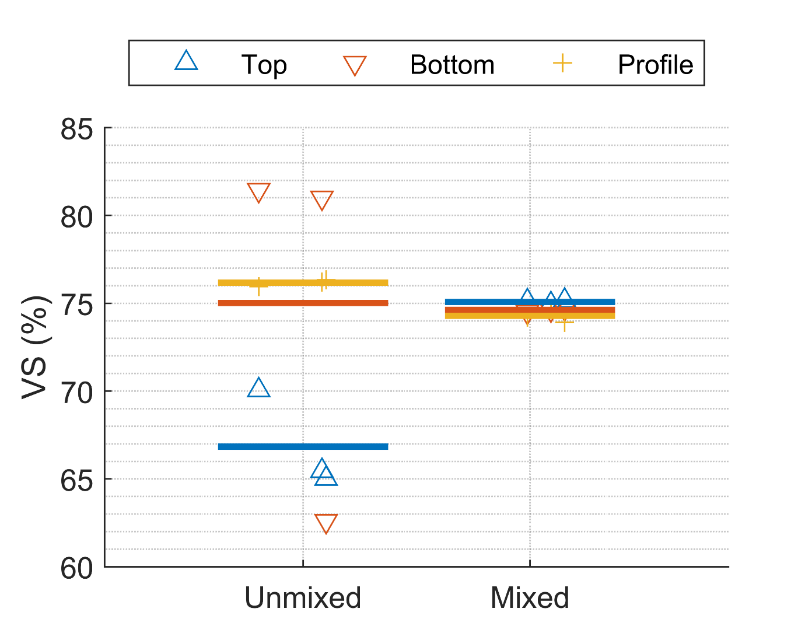


Figure S8. Volatile solids for samples collected at the top, bottom or as a profile from an unmixed or mixed slurry tank. The symbols show individual measurements, and the horizontal line shows the average values.
